# Antimicrobial Resistance Profiles and Characterization of *Escherichia coli* Strains from Cases of Neonatal Diarrhea in Spanish Pig Farms

**DOI:** 10.3390/vetsci7020048

**Published:** 2020-04-21

**Authors:** Anna Vidal, Laia Aguirre, Chiara Seminati, Montse Tello, Noelia Redondo, Marga Martín, Laila Darwich

**Affiliations:** 1 Departament de Sanitat i d’Anatomia Animals, Universitat Autònoma de Barcelona, 08193 Bellaterra, Spain; annavordeig@gmail.com (A.V.); laia.aguirre@uab.cat (L.A.); chiara.seminati@uab.cat (C.S.); montse.tello@uab.cat (M.T.); noelia.redondo@uab.cat (N.R.); 2 IRTA, Centre de Recerca en Sanitat Animal (CReSA, IRTA-UAB), Campus de la Universitat Autònoma de Barcelona, 08193 Bellaterra, Spain

**Keywords:** antimicrobial resistance, ESBL genes, colistin resistance, *Escherichia coli*, neonatal diarrhea, pig

## Abstract

*Escherichia coli* is considered one of the most common agents associated with neonatal diarrhea in piglets. The aim of this work was to characterize the pathogenic and antimicrobial resistance (AMR) profiles of 122 *E. coli* strains isolated from pigs suffering diarrhea (n = 94) and pigs without diarrhea (n = 28) of 24 farms in Spain. Virulence factors, toxins and AMR (ESBL and colistin) genes and AMR phenotypes of *E. coli* isolates were analyzed. Low prevalence of pathogenic *E. coli* strains (26%) was found in both groups. However, ETEC and VTEC strains were more frequently isolated from diarrheic piglets. Irrespectively of diarrhea occurrence, 97.5% of the strains showed a multidrug-resistance (MDR) profile to aminopenicillins, sulfonamides and tetracyclines. It was found that 22% of *E. coli* was CTX-M+, with CTX-M-14 being the principal allelic variant. Remarkably, 81.5% of CTX-M+ strains were isolated from diarrheic animals and presented an extended MDR profile to aminopenicillins, quinolones and aminoglycosides. Finally, low frequencies of colistin resistance genes *mcr*-1 (4/122) and *mcr*-4 (1/122) were found. MDR *E. coli* strains are circulating in pig farms of Spain, representing a serious threat to animal and public health. More appropriate diagnostic approaches (genetic and AMR phenotypic analysis) should be implemented in animal health to optimize antibiotic treatments.

## 1. Introduction

*Escherichia coli* has historically been considered as one of the most common agents associated with diarrhea in suckling and post-weaned piglets [1]. There is high diversity and variants of *E. coli* strains integrating the normal gut microbiota, with most of them being considered not pathogenic [2]. The characterization of pathogenic *E. coli* strains is usually based on the presence of virulence factors [3]. In piglets, *E. coli* pathogenic strains can be classified into different pathotypes: enterotoxigenic (ETEC) strains releasing heat-labile (LT) and heat-stable Sta and Stb exotoxins, intimin (*eae*)-producing enteropathogenic (EPEC) strains and verotoxigenic (VTEC) strains producing VT1/VT2 verotoxins [4].

The routine use of antimicrobials in livestock, especially in the pig industry, for either ‘prophylaxis’ or ‘metaphylaxis’ represents a serious hazard for the selection of multidrug-resistant (MDR) *Enterobacteriaceae* strains [5,6]. In fact, the effectiveness of treatments against *E. coli* is threatened by the dramatic increase of extended-spectrum beta-lactamases (ESBL)-producing isolates worldwide [7,8,9]. A high number of ESBL have been described and designated according to the strains of bacteria or plasmid that produce them by using letters that usually evoke the name of the target antibiotic, but they are also named after substrates, their biochemical properties, location of their discovery and the name of the patient or the discovering investigators [10]. Genes encoding ESBL enzymes are usually located in plasmids and can be easily horizontally transferred among different bacterial populations. Most ESBL derive from the first enzymes discovered, TEM (named after the patient (Temoneira) providing the first sample) and SHV (sulfhydryl reagent variable), which confer resistance to beta-lactams such as ampicillin or amoxicillin. The most recent ESBLs are derived from the CTX-M (active on cefotaxime-Munich) enzymes, which confer resistance to third- and fourth-generation cephalosporins [10,11]. Cephalosporins constitute one of the largest families of antimicrobials widely used in both human and veterinary medicine [12]. On the other hand, in veterinary medicine, colistin sulfate is indicated for the treatment of *Enterobacteriaceae* infections and has been widely used as a preventive mass-medication of colibacillosis in piglets [13]. Although colistin is one of the antimicrobial agents with the lowest rates of resistance, the emergence of the *mcr*-1 (mobile colistin resistant) plasmid in the *Enterobacteriaceae* population has resulted in the appearance of strains with acquired resistance to this antibiotic [14,15]. In order to stop the increase of colistin-resistant strains, the European Medicines Agency established in 2016 that all EU members should restrict and reduce the use of colistin in animals for treating infections with a target level of 5 mg/PCU, where PCU refers to the ‘population correction unit’ and takes into account the animal population as well as the estimated weight of each particular animal at the time of treatment with antimicrobials. [16].

During recent decades, the importance of *E. coli* in porcine neonatal diarrhea seems to have decreased [17], possibly due to the successful vaccination plans implemented in the pig farms [18]. Therefore, antimicrobial treatment might not be required in most cases of diarrhea, especially in those where the role of the *E. coli* is not well defined. However, the systematic mass-medication of piglets suffering from diarrheic processes is still common in many cases.

The aim of the present work was to characterize the virulence factors and to determine the genotypic and phenotypic antimicrobial resistance patterns of *E. coli* isolates from cases of neonatal diarrhea in conventional pig farms of Catalonia (Spain). Moreover, in order to assess the clinical relevance of all isolates, samples from non-diarrheic pen-mates were also analyzed and compared to the clinical cases.

## 2. Materials and Methods

### 2.1. Microbiological Testing

A total of 122 *E. coli* isolates obtained from 24 conventional Spanish farms suffering neonatal diarrhea outbreaks during 2017 and 2018 were recovered from a previous study [19]. All the studied farms were located in Catalonia (NE of Spain), the Spanish region with the greatest number of production farms and one of the highest pig-density (242 animals/km^2^) regions in Europe. The sampling procedure included 5–10 samples from diarrheic animals and 3–5 samples from apparently healthy pen-mates for each farm. One gram of fecal sample was obtained directly from the animals using rectal swabs and submitted for diagnostic testing to the Laboratori Veterinari de Diagnosi de Malalties Infeccioses of the Universitat Autònoma de Barcelona (Spain). Besides *E. coli*, all samples were tested for a panel of enteric infectious agents which comprised *C. perfringens* types A and C toxins (Cpα, Cpβ, Cpβ2); *C. difficile* toxins (TcdA, TcdB); rotavirus A (RVA), B (RVB) and C (RVC); porcine epidemic diarrhea virus (PEDV) and transmissible gastroenteritis virus (TGEV), and these general results were described elsewhere [19].

For *E. coli* isolation, stool samples were cultured in standard Blood Agar (BD GmbH, Germany) and MacConkey Agar (Oxoid, UK) and aerobically incubated at 37 °C for 24 h. A total of 122 pure isolates were collected: 94 from diarrheic samples of 1-week-old diseased piglets and 28 from feces of apparently healthy pen-mates. All strains were confirmed as *E. coli* using conventional biochemical tests. Only *E. coli* strains obtained as pure cultures from a direct seeding of feces were used for the characterization, since the lack of microbial diversity was considered abnormal.

### 2.2. Characterization of E. coli Virulence Factors and Toxins

The presence of *E. coli* toxins (Sta, Stb, LT, EAST1, VT1 and VT2), fimbrial adhesins (F4, F5, F6, F18 and F41) and non-fimbrial adhesin *eae* genes was analyzed by qualitative PCR, as described by Toledo et al. (2012) with slight modifications [20]. Briefly, PCR assays were carried out using the Biotaq DNA polymerase kit (Biotaq, Ecogen) using previously described primers [19]. The final 25 µL mixture consisted of: 1× PCR Buffer, 0.8 mM of the dNTP mix, 3 mM of MgCl_2_, 1 mM of each primer and 1 U of Taq polymerase. As template, 2.5 µL of the DNA sample was used. The PCR program consisted of 5 min at 94 °C, followed by 30 cycles of 1 min at 94 °C, 1 min of annealing at 57 °C and 1 min of extension at 72 °C, and a final extension step of 7 min at 72 °C. Positive and negative (ultrapure water) controls were included in each run and particular care was taken to prevent carry-over contamination.

Reference *E. coli* strains used as positive controls were FV12048 (F4+ LT+ EAST1+), FV9722 (F5+ F41+), FV7633 (F6+), FV12047 (F18+ Sta+ Stb+) and O157-84 (*eae*+ VT1+ VT2+) were kindly donated by Dr. Blanco (*E. coli* Reference Laboratory, Santiago de Compostela, Spain).

### 2.3. Antimicrobial Resistance Phenotyping Analyses

Antimicrobial susceptibility of *E. coli* isolates was tested using the disk diffusion method [21]. Briefly, one to four colonies were suspended in 5 mL of distilled sterile water to achieve a turbidity of 0.5 in the McFarland scale. The dilution was then seeded onto Mueller–Hinton (Oxoid, UK) plates. Each isolate was tested for the following antimicrobial groups, using commercial disks: aminopenicillins (ampicillin, amoxicillin and amoxicillin/clavulanic acid); cephalosporins (ceftiofur, cephalexin, cefquinome, ceftriaxone); quinolones (ciprofloxacin, enrofloxacin, flumequine); aminoglycosides (gentamicin, neomycin, streptomycin, apramycin); tetracyclines (tetracycline, doxycycline); sulfonamides (sulfonamide, sulfamethoxazole/trimethoprim); florfenicol; colistin; lincospectin. Concentration of the commercial disks and breakpoints for each antimicrobial are shown in Table 1.

Additionally, minimum inhibitory concentration (MIC) test was performed in order to evaluate antimicrobial susceptibility to colistin, using the broth microdilution method in 96-well plates [22]. *E. coli* ATCC 25,922 was used as the quality control strain. Briefly, the tested colistin concentrations ranged from 0.25 to 8 μg/mL. The tested strains were considered resistant when their MIC value was higher than the wild type cut-off value (MIC > 2μg/mL) [23].

### 2.4. Genetic Characterization of Antimicrobial Resistance Genes

Molecular diagnosis of AMR genes was performed for the ESBL genes *bla*_SHV_, *bla*_CTX-M_, *bla*_CMY1_, *bla*_CMY2_ and *bla*_TEM_; carbapenemase *bla*_OXA_ and *mcr*-1 to *mcr*-5 colistin-resistance genes [25]. PCR conditions were homogenized for all the reactions as follows: 5 min at 94 °C, followed by 25 cycles of 1 min at 94 °C, 1 min of annealing at 55 °C and 1 min of extension at 72 °C, and a final extension step of 7 min at 72 °C. Amplified PCR products were Sanger sequenced for verification at the Genomic and Bioinformatics Service of the Universitat Autònoma de Barcelona (Barcelona, Spain). Sequences and chromatograms were manually explored to trim bad-quality bases using the BioEdit software. The obtained sequences were aligned using Clustal Omega and blasted against those described at the National Center for Biotechnology Information (NCBI) database.

### 2.5. Statistical Analysis

Chi-squared or Fisher exact tests were used for comparison between proportions when appropriate. Results were considered statistically significant when *p* < 0.05.

## 3. Results

### 3.1. Microbiological Identification and Characterization of E. coli Isolates

Most of the *E. coli* isolates presented a very low prevalence of virulence factors and toxins (<5%). with the exception of EAST1, which was present in more than 70% of the strains (Table 2). Moreover, 74% of the strains were negative for all toxins and intimin (*eae*) genes. As such, it was not possible to classify them into any pathotype.

Table 3 shows the number of isolates presenting virulence factors and toxins grouped by pathotype and their total frequency in diarrheic and not diarrheic cases. The most common pathotypes within the classified strains were EPEC (n = 17) and ETEC (n = 15), with the frequency of EPEC being two times higher (21% vs. 10.6%) in non-diarrheic piglets. As expected, the frequency of ETEC was higher in animals presenting diarrhea than in those without clinical signs (15% vs. 3.5%). The presence of VTEC (4.3%) was only detected in cases of diarrhea. Similarly, *E. coli* strains positive for F4, LT, Sta, VT1 and VT2 genes were only isolated from clinical cases.

### 3.2. Antimicrobial Resistance Phenotyping and Genotyping

According to the results obtained by the disk diffusion method, the overall prevalence of AMR in *E. coli* isolates was very high for most of the nine tested antimicrobial classes. The highest rates of resistance were found for sulfonamides (87.3%), tetracyclines (87.3%) and aminopenicillins (80.1%), followed by quinolones (49%), cephalosporins (32.9%) and aminoglycosides (27.9%). By contrast, low resistance levels were observed for colistin (6.5%) and apramycin (5.9%). Regarding the MIC results, 6 out of 122 (5%) were resistant to colistin (MIC > 2 μg/mL): 5 of them had an MIC value of 4 μg/mL, and one isolate had an MIC of 8 μg/mL. Comparing the colistin results obtained by disk diffusion and the MIC, only one strain was considered resistant to colistin by both methods.

Most of the strains, 97.5% (119/122), presented a multidrug-resistance (MDR) profile (≥3 antimicrobial categories), and 67.2% of them offered resistance to six antibiotic classes. Moreover, 30% of the strains (37/122) showed an extensive drug-resistance (XDR) profile (>7 antimicrobial classes). Two strains presented resistance to all the tested antimicrobials except for colistin.

**Figure 1:**
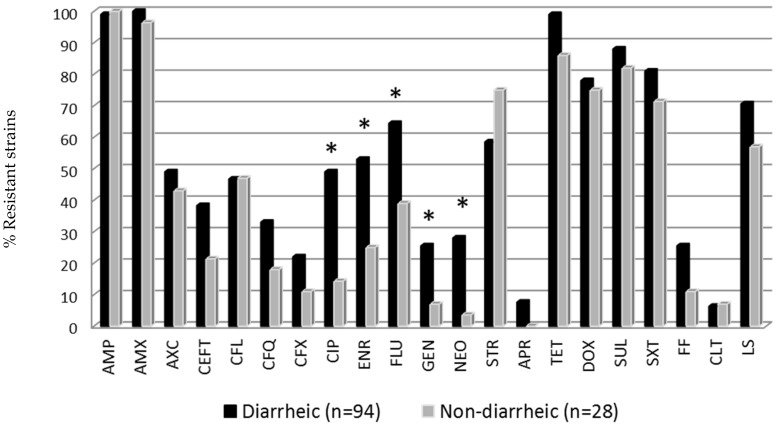
Antimicrobial resistance of *E. coli* strains from diarrheic and non-diarrheic groups. * Statistical differences between diarrheic and non-diarrheic animals (Fisher exact test *p* < 0.05). Antibiotic abbreviations are shown in Table 1.

The comparison study between *E. coli* isolates from diarrheic and non-diarrheic samples showed statistical differences in the proportion of AMR *E. coli* isolates (Figure 1). Remarkably, piglets presenting diarrhea had higher prevalence of *E. coli* strains resistant to quinolones and aminoglycosides (gentamicin and neomycin) compared to the non-diarrheic isolates (Fisher exact test *p* < 0.05).

The most prevalent ESBL gene was the *bla*_CTX-M_ found in 22% (27/122) of the total samples, and 81.5% (22/27) of them were isolated from diarrheic animals. All the CTX-M+ strains except one were successfully sequenced and could be classified into the following *bla*_CTX-M_ genotypes: CTX-M-14 (38.5%), CTX-M-1 (19.2%), CTX-M-15 (19.2%), CTX-M-32 (11.5%), CTX-M-27 (7.7%) and CTX-M-3 (3.8%).

**Figure 2:**
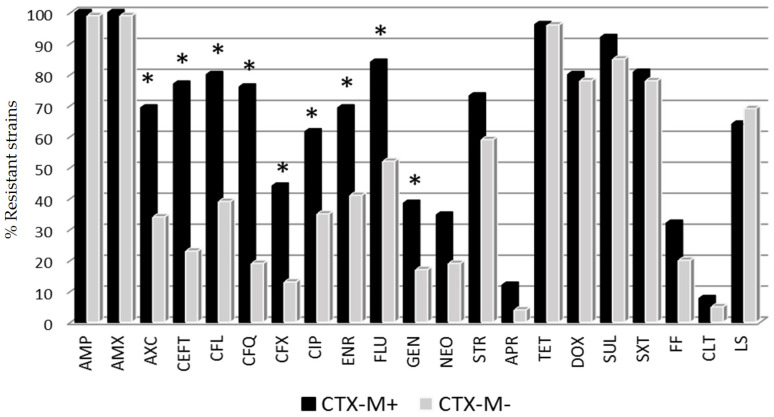
Percentage of *E. coli* resistant strains to different antimicrobial categories according to CTX-M gene expression. * Statistical differences between CTX-M+ (positive) and CTX-M– (negative) strains (Fisher exact test *p* < 0.05). Antibiotic abbreviations are shown in Table 1.

On the other hand, *E. coli* harboring CTX-M+ genes presented a wider resistance pattern than those CTX-M-negative strains (CTX-M–). Specifically, 81.5% of the CTX-M+ strains showed resistance to more than 10 antimicrobials, while in the case of CTX-M– the percentage was 37.9%. Statistical differences between CTX-M+ and CTX-M– strains were found for amoxicillin/clavulanic acid, cephalosporins, quinolones and gentamicin drugs (Figure 2). By contrast, colistin resistance genes were detected in a low proportion of cases: four strains harbored the *mcr*-1 gene (3%), and one strain was positive to *mcr*-4. Only one of the *mcr-1*+ strains, isolated from a diarrheic animal, exhibited phenotypic resistance to colistin in the MIC test (8 μg/mL).

## 4. Discussion

The aim of this work was to characterize the *E. coli* strains involved in porcine neonatal diarrhea outbreaks by genotyping and phenotyping isolates grown as pure cultures from both clinical cases of diarrheic and non-diarrheic healthy pen-mates.

Surprisingly, the general prevalence of strains encoding genes for virulence factors, as well as the presence of the typical *E. coli* pathotypes, was low in our pig population studied. Merely 26% (32/122) of the strains could be classified into a specific pathotype. EPEC was the most frequent one, representing 14% of the total isolates, followed by ETEC (12%) and VTEC (3%). The presence of EPEC is usually reported as a causative agent of diarrhea, principally in post-weaned but also in suckling pigs, but in this study, the presence of this pathotype was found more prevalent in non-diarrheic piglets (21.4%) than in the diarrheic ones (11.8%). Similar results have been reported in previous works in agreement with the results presented in the present study. Mainly, EPEC prevalence ranging from 3% to 26% in diarrheic animals and 9% to 17% in non-diarrheic animals has been described [26,27,28]. Finally, EAST1 gene was highly detected in both diarrheic (70%) and non-diarrheic groups (86%) but again with no relationship with the clinical onset. The EAST1 toxin is produced by several *E. coli* pathotypes [29], and although initially it was believed to be involved in the pathogenesis of diarrhea, most authors have concluded that this toxin may not be a virulence determinant, because it has been commonly isolated from healthy animals as well [30,31].

On the contrary, ETEC and VTEC were more frequently isolated from diarrheic samples, suggesting a possible association between these pathotypes and the emergence of disease in 1-week-old piglets.

The AMR phenotypes of the *E. coli* isolates showed a wide resistance to sulfonamides, tetracyclines and aminopenicillins in both diarrheic and non-diarrheic piglets. High percentages of antimicrobial resistance for amoxicillin and ceftiofur have been previously reported in pig farms from Catalonia [32]. However, other studies conducted in other regions of Spain [33] or in Ireland [34] reported lower levels of resistance to tetracycline, sulfamethoxazole/trimethoprim, ampicillin and streptomycin. These differences could be explained by the fact that Catalonia is one of the areas with greater density of pigs in Europe and therefore is likely subjected to a high consumption of antibiotics.

On the other hand, an interesting finding of this study was that *E. coli* isolates from diarrheic cases additionally presented extended AMR to quinolones and aminoglycosides (gentamicin and neomycin) compared to those from healthy pigs. These findings agree with other studies performed in Ontario [35] and in Denmark [36], which found consistently higher frequencies of resistance in diarrheic and ETEC isolates, respectively.

The genotyping study showed that the *E. coli* strains containing the *bla*_CTX-M_ gene presented resistance to third- and fourth-generation cephalosporins, amoxicillin/clavulanic acid, quinolones and aminoglycosides. The relationship between ESBLs and quinolone resistance mechanisms has been documented by several authors [37,38,39], and it has been suggested that ESBL genes and plasmid-mediated quinolone resistance are located within the same plasmid [40]. Furthermore, strains harboring the *bla*_CTX-M_ gene presented higher values of phenotypical resistance than those without this gene, mainly to all the tested beta-lactams, quinolones and gentamicin.

Regarding the genotyping of the CTX-M+ strains, all the allelic variants described in this study have previously been reported in pigs, with CTX-M-14 also being the most prevalent [41,42]

In the case of colistin, resistance was assessed using two different methods, the disk diffusion method and the MIC test, finding low concordance between both techniques. According to several authors, the disk diffusion method does not seem to be a reliable method for testing the efficacy of colistin due to the poor diffusion of polymyxins in agar. Therefore, the use of other tests, such as MIC, is more adequate for testing the susceptibility of colistin [43].

In general, the prevalence of porcine colistin-resistant *E. coli* strains reported in Europe is low: 0.9% in Poland [44], 1.5% in France [45], 2.9% in Germany [46] and 3.7% in Switzerland [47]. These results are in concordance with the 5% of prevalence obtained in this study using the MIC test. Nevertheless, García et al. (2018) have reported an extremely high prevalence of colistin resistance (77%) in the northwest area of Spain [48]. Variations in animal health management, biosecurity and sanitation procedures in pig production between different regions of the same country could explain this different colistin-resistance prevalence.

Likewise, the prevalence of *E. coli* isolates harboring the *mcr*-1 gene was low (3%) in this study, in agreement with results reported in Germany [46,49] and Belgium [50] with prevalence below 10%. Nevertheless, prevalences of 26% and 88% have been published in studies conducted in the north-west area of Spain [48] and in France [51], respectively. Only one *E. coli* isolate of this study presented the *mcr*-4 gene; however, the frequency of this gene in the Spanish pig farms is variable, ranging from 21% [8] to 73% [48] depending of the region. Regarding other *mcr* genes, none of the isolates of this study were positive for *mcr*-2, *mcr*-3 or *mcr*-5. The *mcr*-2 gene has been detected in porcine and bovine *E. coli* isolates from Belgium [50], *mcr*-3 in *E. coli* isolates from pigs in UK [52] and *mcr*-5 in 3.4% of isolates in Spain [48].

In summary, *E. coli* CTX-M+ strains isolated from diseased piglets showed antimicrobial multi-resistance to β-lactams, tetracyclines, sulfonamides and lincospectin extended to quinolones and aminoglycosides. This high prevalence of *E. coli* MDR strains in the pig population can represent a serious threat for animal and human health [53].

## 5. Conclusions

More appropriate diagnostic approaches, including genetic and phenotypic analysis of AMR profiles, should be implemented in animal health to optimize the use of antibiotics for treating diseased animals when necessary and with an effective antimicrobial agent, as the presence of highly resistant strains harboring ESBL and *mcr* genes circulating in pig farms can represent a potential threat to animal and human health.

## Figures and Tables

**Table 1:** Concentrations and breakpoints of the antibiotic disks used for the disk diffusion method.

Antibiotic ^a^	Concentration (µg/mg) (Brand, Country)	Breakpoint (mm)		Reference ^b^
		S	R	
Amoxicillin (AMX)	25 (BD, USA)	≥17	≤13	CLSI M100; human [21]
Amoxicillin/clavulanic (AXC)	20/10 (Oxoid, UK)	≥18	≤13	CLSI M100; human [21]
Ampicillin (AMP)	10 (BD, USA)	≥17	≤13	CLSI VET08; dog [22]
Ceftiofur (CEFT)	30 (BD, USA)	≥21	≤17	CLSI VET08; cattle *E. coli* and swine *Salmonella* Cholerasuis [22]
Cephalexin (CFL)	30 (Oxoid, UK)	≥18	≤14	CLSI VET08; dog [22]
Cefquinome (CFQ)	10 (Conda Lab, Spain)	≥21	≤17	CLSI VET08 [22]
Ceftriaxone (CFX)	30 (BD, USA)	≥21	≤13	CLSI VET08 [22]
Ciprofloxacin (CIP)	5 (BD, USA	≥21	≤15	CLSI M100; human [21]
Enrofloxacin (ENR)	5 (BD, USA)	≥23	≤16	CLSI VET08; dog, cat and poultry [22]
Flumequine (FLU)	30 (BD, USA)	≥25	<21	EUCAST [23]
Gentamicin (GEN)	10 (BD, USA)	≥16	≤12	CLSI VET08: dog, horse [22]
Neomycin (NEO)	30 (BD, USA)	≥17	≤12	CLSI VET08 [22]
Streptomycin (STR)	10 (BD, USA)	≥15	≤11	CLSI M100; human [21]
Apramycin (APR)	15 (Oxoid, UK)	≥15	≤10	CLSI VET08 [22]
Tetracycline (TET)	30 (BD, USA)	≥15	≤11	CLSI M100; human [21]
Doxycycline (DOX)	30 (Oxoid, UK)	≥16	≤12	CLSI VET08; horse [22]
Sulfonamide (SULF)	300 (Oxoid, UK)	≥17	≤12	EUCAST [23]
Sulfamethoxazole/trimethoprim (SXT)	23.75 + 1.25 (BD, USA)	≥16	≤10	CLSI VET08 [22]
Florfenicol (FF)	30 (Oxoid, UK)	≥22	≤18	CLSI VET08; swine [22]
Colistin (CLT)	50 (BD, USA)	≥18	<15	CA-SFM, veterinary ^c^ [24]
Lincospectin (LS)	2 (Oxoid, UK)	≥20	≤16	EUCAST [23]

^a^ Antibiotic and abbreviations used also in Figure 1 and Figure 2. ^b^ CLSI veterinary breakpoints were preferably used. If not available, CLSI human, EUCAST or CA-SFM veterinary breakpoints were used. ^c^ The measurement of the minimum inhibitory concentration of colistin in broth dilution remains the reference method.

**Table 2:** Comparison of virulence factors and pathogenic *E. coli* prevalence in diarrheic (n = 94) and non-diarrheic (n = 28) pig groups.

Virulence Factors/Toxins ^1^	Diarrheic n = 94	Non-Diarrheic n = 28
	n (%)	n (%)
LT	2 (2.1)	0
Sta	4 (4.3)	0
Stb	12 (12.8)	1 (3.5)
EAST1	66 (70.2)	24 (85.7)
VT1	1 (1.1)	0
VT2	3 (3.2)	0
F4	4 (4.3)	0
F5	0	0
F6	0	0
F18	1 (1.1)	1 (3.5)
F41	3 (3.2)	1 (3.5)
***eae***	11 (11.8)	6 (21.4)

^1^*E. coli* toxins (LT, Sta, Stb, EAST1, VT1 and VT2), fimbrial adhesins (F4, F5, F6, F18 and F41) and non-fimbrial adhesin *eae* genes.

**Table 3:** Description of the profiles of virulence factor production and classification of the toxigenic strains into pathotypes of *E. coli* strains.

Pathotype ^1^	Diarrheic (n = 94)	Non-Diarrheic (n = 28)	Total (n = 122)
**ETEC (n = 11)**			
Stb+, EAST1+	6	1	7
Stb+, EAST1+, Sta+	1	0	1
Stb+, EAST1+, Sta+, F4+	2	0	2
LT+	1	0	1
LT+, EAST1+ Total n (%)	1 11 (11.7%)	0 1 (3.5%)	1 12 (9.8%)
**ETEC/EPEC (n = 1)**			
Stb+, *eae*+, EAST1+, F41+ Total n (%)	1 1 (1.1%)	0 0	1 1 (0.8)
**EPEC (n = 16)**			
*eae*+	1	0	1
*eae*+, EAST1+	7	5	12
*eae*+, EAST1+, F41+	1	1	2
*eae*+, EAST1+, F18+ Total n (%)	1 10 (10.6%)	0 6 (21%)	1 16 (13.1%)
**VTEC (n = 1)**			
VT1+ Total n (%)	1 1 (1.1%)	0 0	1 1 (0.8)
**VTEC/ETEC (n = 3)**			
Stb+, VT2+	1	0	1
Stb+, VT2+, EAST1+	1	0	1
Stb+, VT2+, EAST1+, Sta+, F4+ Total n (%)	1 3 (3.2%)	0 0	1 3 (2.5%)

^1^ Enterotoxigenic (ETEC) strains releasing heat-labile LT and heat-stable Sta and Stb exotoxins; intimin (*eae*)-producing enteropathogenic (EPEC) strains; verotoxigenic (VTEC) strains producing VT1/VT2 verotoxins.
